# Invasive and Non-Invasive Diagnostic Pathways in the Diagnosis of Cardiac Amyloidosis

**DOI:** 10.3390/jcdd10060256

**Published:** 2023-06-11

**Authors:** Alexandros Briasoulis, Dimitrios Bampatsias, Adamantia Papamichail, Toshiki Kuno, John Skoularigis, Andrew Xanthopoulos, Filippos Triposkiadis

**Affiliations:** 1 Amyloidosis Center, Department of Clinical Therapeutics, Faculty of Medicine, Alexandra Hospital, National and Kapodistrian University of Athens, 80 Vasilissis Sophias Avenue, 11528 Athens, Greece; 2 Montefiore Medical Center, Bronx, NY 10467, USA; 3 Department of Cardiology, University Hospital of Larissa, 41110 Larissa, Greece

**Keywords:** cardiac amyloidosis, diagnosis, AL, ATTR, endomyocardial biopsy

## Abstract

The appropriate diagnosis and subtyping of cardiac amyloidosis (CA) is frequently missed or delayed due to its vague presentation, clinical overlapping, and diagnostic pitfalls. Recent developments in both invasive and non-invasive diagnostic techniques have significantly changed the diagnostic approach of CA. With the present review, we aim to summarize the current diagnostic approach of CA and to underline the indications of tissue biopsy, either surrogate site or myocardial. The most important factor for timely diagnosis is increased clinical suspicion, especially in certain clinical scenarios. Appropriate imaging with echocardiography or cardiac magnetic resonance (CMR) can provide significant evidence for the diagnosis of CA. Importantly, all patients should undergo monoclonal proteins assessment, with these results significantly determining the steps to follow. A negative monoclonal protein assessment will lead to a non-invasive algorithm which, in combination with positive cardiac scintigraphy, can establish the diagnosis of ATTR-CA. The latter is the only clinical scenario in which the diagnosis can be established without the need of biopsy. However, if the imaging results are negative but the clinical suspicion remains high, a myocardial biopsy should be performed. In the case of the presence of monoclonal protein, an invasive algorithm follows, first by surrogate site sampling and then by myocardial biopsy if the results are inconclusive or prompt diagnosis is needed. The role of endomyocardial biopsy, even though limited by current advances in other techniques, is highly valuable in selected patients and is the only method to reliably establish a diagnosis in challenging cases.

## 1. Introduction

Amyloidosis is a group of multisystemic infiltrative diseases, often affecting the heart, which are caused by extracellular amyloid fibrils deposition [1]. Forty-two amyloidogenic proteins have been identified to date [1,2,3,4], but the two most common subtypes affecting the heart, accounting for more than 95% of all cases, are transthyretin (ATTR) and light chain (AL) amyloidosis [2]. ATTR is caused either by age-related misfolding of normal transthyretin (wild type ATTR, wtATTR) or amyloidogenic mutations in the TTR gene (hereditary ATTR, hATTR), while misfolded immunoglobulin light chains result in AL [5]. Cardiac amyloidosis (CA) is a serious, often underdiagnosed [6] condition causing heart failure, usually with preserved ejection fraction. Notably, CA presentation is not specific, including symptoms of heart failure, valvular disease, rhythm conduction disturbances, and commonly, but not exclusively, preserved ejection fraction [7,8,9,10,11]. In rare cases, patients with systemic amyloidosis may have asymptomatic cardiac involvement [9]. Early diagnosis and subtyping of CA is of paramount importance, since AL amyloidosis is characterized by a grim prognosis [12,13], and early treatment initiation is crucial to halt ATTR-CA progression [5,14]. Recent advances in non-invasive techniques have led to their integration in the proposed diagnostic algorithms of CA, but tissue biopsy remains the gold standard for diagnosing and typing of amyloidosis. Currently, only ATTR can be diagnosed non-invasively, as long as certain presumptions are met [5,14]. However, due to the non-specific presentation and phenotypical overlapping between the subtypes of CA, there are diagnostic pitfalls, resulting in a significant delay to establishing a diagnosis. In the present review, we aim to summarize the contemporary diagnostic approach of CA and to highlight the role of tissue and myocardial biopsy in the accurate and timely diagnosis of CA.

## 2. Diagnostic Algorithm

### 2.1. Clinical Suspicion

The most important factor for CA diagnosis is clinical suspicion prior to diagnostic work-up initiation. Even though CA is considered a rare disease, it should always be considered in differential diagnoses in certain clinical scenarios due to its high rates of mortality and morbidity [5,14]. These clinical scenarios include the presence of the following “red flag” cardiac and/or extracardiac signs and symptoms, usually in a patient with increased left ventricular wall thickness (>12 mm): (1) heart failure >65 years; (2) aortic stenosis >65 years; (3) hypotension, or normotensive in a previously hypertensive patient; (4) peripheral neuropathy; (5) autonomic dysfunction; (6) bilateral carpal tunnel syndrome; (7) biceps tendon rupture; (8) lumbar spinal stenosis; (9) proteinuria; (10) purpura; (11) macroglossia; (12) pseudo-infarct pattern in ECG; (13) low/decreased QRS voltage compared to degree of left ventricular hypertrophy; (14) atrio-ventricular conduction disease; (15) decreased global longitudinal strain with apical sparing on echocardiography; (16) subendocardial late gadolinium enhancement and/or increased extracellular volume on cardiac magnetic resonance imaging; (17) significantly elevated NT-pro-BNP, disproportionate to degree of HF; (18) unexplained persistent low-level cardiac troponin elevation; and (19) family history. Table 1 includes the most common “red flag” clinical scenarios for CA and each subtype. The presence of any of the above should lead to a thorough medical history and clinical examination, seeking other “red flag” symptoms which could increase the likelihood of amyloidosis [5,13,14]. In addition, cardiac disease in the setting of plasma cell dyscrasia, nephrotic syndrome, and chronic systemic inflammatory condition should raise the intuition of CA, especially if suspicious cardiac imaging findings are evident.

### 2.2. Cardiac Biomarkers

Cardiac biomarkers are extremely important in the diagnosis of cardiac amyloidosis. First, persistently elevated troponin and/or disproportionally elevated NT-proBNP compared to HF clinical presentation should raise the suspicion of CA [15]. Both are often increased in the early stages of CA, even before symptom onset or echocardiographic evidence of cardiac involvement, highlighting the cytotoxic properties of amyloid fibrils on myocardial cells [16,17,18]. Cardiac troponins, especially high-sensitivity (hs) troponins, and natriuretic peptides are usually higher in AL compared to ATTR-CA, reflecting the greater cytotoxic properties of light-chains-derived amyloid fibrils [19,20]. Regarding ATTR, cardiac biomarkers are more often higher in wild type ATTR than in variant ATTR, likely due to increased age and impaired renal function. In patients with variant ATTR, levels of cardiac biomarkers can vary significantly based on the underlying mutation and the degree of cardiac involvement [20]. NT-proBNP and troponin measurement is currently recommended as the first steps of the diagnostic algorithm of CA. A recent study has suggested that NT-proBNP < 180 ng/L and hs-TnT < 14 ng/L can reliably exclude the diagnosis of CA [21], showing that low levels of circulating biomarkers could be used safely to exclude CA and avoid further invasive and/or non-invasive testing. Biomarkers assessment should be combined with clinical and imaging parameters at the early phases of the diagnostic algorithm of systemic amyloidosis to support further decision making [22].

### 2.3. Monoclonal Protein Assessment

Upon raised suspicion for CA, definitive diagnosis should be established as soon as possible, since patient outcomes rely on early treatment initiation, in particular in AL-CA [5,14]. Since AL and ATTR are responsible for 95% of CA cases, the diagnostic work-up focuses on differentiating between these subtypes of CA. All patients with suspected CA should undergo assessment for the presence of monoclonal protein in serum and urine, by serum protein electrophoresis with immunofixation (SPIE), urine protein electrophoresis with immunofixation (UPIE) and serum kappa/lambda free light chains ratio [13,23,24]. Serum kappa/lambda light chains ratio is considered normal between 0.26–1.65, by Freelite assay, and 0.53–1.51 by N latex assay. Monoclonal lambda gammopathy is indicated by a kappa/lambda ratio < 0.26, whereas monoclonal kappa gammopathy is indicated by a kappa-lambda ratio > 1.65 [25]. Importantly, kidney disease can cause elevation in the kappa/lambda ratio, a fact that should be taken into consideration, particularly in patients with eGFR ≤ 45 mL/min/1.73 m^2^. If monoclonal protein is detected, a hematologist should be consulted for further evaluation, while negative SPIE, UPIE and normal kappa/lambda ratio have a negative predictive value of 99% for AL [26]. Interestingly, current literature suggests that a significant percentage of patients with suspected CA do not undergo complete evaluation for monoclonal proteins [27].

### 2.4. Imaging

**Figure 1:**
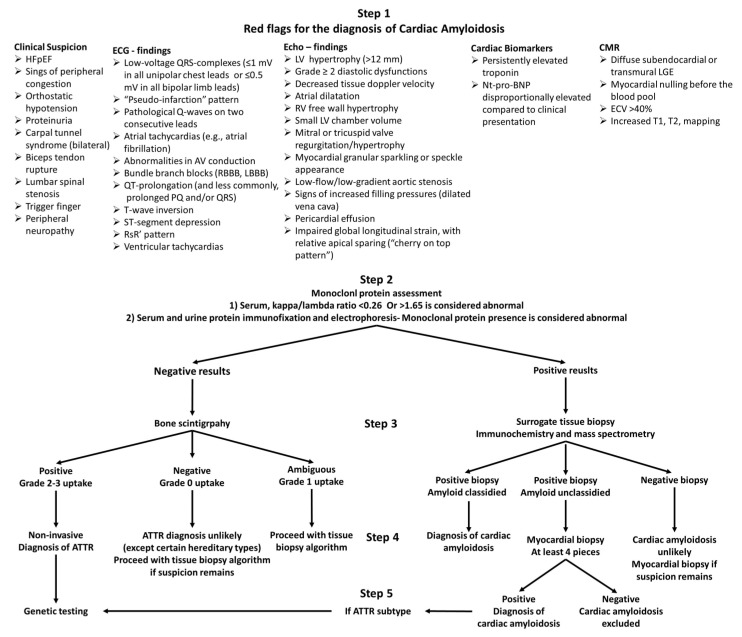
Diagnostic algorithm of amyloid cardiomyopathy. Abbreviations: HFpEF = heart failure with preserved ejection fraction, RBBB = right bundle branch block, LBBB = left bundle branch block, LV = left ventricle, NT-proBNP = N-terminal pro-brain natriuretic peptide, LGE = late gadolinium enhancement, ECV = extracellular volume, ATTR = transthyretin amyloidosis.

The appropriate laboratory assessment should always be conjoined with cardiac imaging, including echocardiography and/or cardiac magnetic resonance (CMR) and, in most cases, bone scintigraphy. Imaging findings suggestive of CA are shown in Figure 1.

#### 2.4.1. Echocardiography

Overall, hypertrophy is more apparent in ATTR than AL amyloidosis and is more pronounced compared to hypertensive or valvular heart disease [28]. Even in the late stages of CA, the LVEF may still be normal, but the indexed stroke volume is typically significantly decreased. Impaired global longitudinal strain (GLS) is suggestive of CA (GLS < −13%), while a characteristic pattern is observed with apical sparing (“cherry-on-top” pattern), and apical GLS/basal GLS > 2.9 is suggestive of CA [5,14]. Strain analysis can reveal abnormal cardiac mechanics before changes in LVEF or other parameters of myocardial structure. In a recent study, the LVEF strain ratio (LVEF/absolute value of GLS), was found to be significantly higher in CA patients compared to individuals with myocardial hypertrophy or healthy controls [29]. In the LA appendage, atrial wall thickening, dilatation, and thrombosis can be seen, while LA function is reduced [29]. Other echocardiographic findings include atrial dilatation, right ventricular free wall hypertrophy, small LV chamber volume [6], dysfunction of the mitral or tricuspid valves [30], myocardial granular sparkling (in non-harmonic imaging) or speckle appearance (in harmonic imaging), thickening of atrio-ventricular (AV) valves and atrial septum, as well as low-flow, low-gradient aortic stenosis, and signs of increased filling pressures (dilated vena cava, small pericardial effusion) [9,30,31,32,33,34,35].

Speckle-tracking echocardiography (STE) can be used to assess CA. According to a recent study [26], patients with unexplained LV hypertrophy who had peak left atrial longitudinal strain (LA-PALS) or LA-peak atrial contraction strain (PACS) in the first quartile were up to 8.76 times more likely to develop CA than individuals without these conditions. STE also enables accurate investigation of the reservoir and active contraction functions of the left atrium, both of which are compromised in CA patients compared to healthy controls, independent of left atrial size. LA strain is markedly reduced in CA patients, particularly in those with transthyretin amyloidosis (ATTR-CA). LA-PALS and LA-PACS are the most effective STE measurements for identifying CA and ATTR-CA [36].

The same study found that LA-PALS and LA-PACS correlated only with E/e′ and LAVI in AL-CA patients, while the observed associations were weaker in ATTR-CA patients [36]. In AL-CA patients, LA-PALS and LA-PACS also correlated with serum NTproBNP and hs-TnT. In contrast, RA-PALS is lower in CA patients, particularly in ATTR-CA patients, than in those without CA. LA-PALS is the only STE parameter to show a connection with CA and ATTR-CA independently of Model 1 and IWT score, and has been linked to both CA and ATTR-CA in patients with unexplained hypertrophy. LA-PACS is also independently related to ATTR-CA. The LA strain is therefore a useful indicator of diastolic function and a potential screening tool for individuals with unexplained hypertrophy. Additionally, the combination of LA-PALS < 6.65% or LA-PACS < 3.62% has independent diagnostic significance and may serve as potential cut-offs for diagnosing ATTR-CA [36].

Although echocardiography remains a primary imaging method for patients with CA-induced heart failure, diagnosing CA is often delayed or missed due to the limited sensitivity and specificity of currently used diagnostic criteria, which focus on wall thickness alone or in combination with NT-proBNP. The diagnostic accuracy of various echocardiographic parameters or their combination in patients with suspected CA remains unknown, as studies have primarily focused on single variables and small retrospective cohorts.

In patients with systemic AL amyloidosis, structural characteristics, such as concentric hypertrophy (PWTd, IVSd, RWT), reduced longitudinal strain (LS), and elevated plasma NT-proBNP are independently associated with the best diagnostic accuracy. However, current diagnostic criteria recommend using wall thickness alone or in combination with NT-proBNP, which are sensitive but not very specific markers. Recently two multiparametric scores, including echocardiographic findings, have been proposed for the diagnosis of CA, but have not been prospectively validated yet [5,37]. The most accurate diagnostic scores for identifying CA in individuals with systemic AL amyloidosis and increasing wall thickness include the AL score (RWT, E/e′, LS, TAPSE) and the IWT score (RWT, E/e′, LS, TAPSE, septal apical-to-base (SAB)), respectively [37]. Points are assigned to variables that meet the ideal diagnostic threshold, resulting in final scores ranging from 0 to 6 for the AL score and 0 to 10 for the IWT score. The particular logistic regression model incorporating relative wall thickness (RWT), E/e′ wave ratio, LS, and tricuspid annular plane systolic excursion (TAPSE) demonstrates the most effective diagnostic performance for AL-CA. Moreover, the addition of SAB ratio improves diagnostic accuracy in patients with increased heart wall thickness. Importantly, changes in various echocardiographic parameters are associated with increased extracellular volume (ECV) values, representing increased myocardial amyloid load [37]. This method allows categorizing specific changes in echocardiography variables according to their likelihood of becoming abnormal at low or high disease burden, as it is determined by ECV, a reliable marker of cardiac infiltration/burden. These two scores, based on specific functional and structural criteria, provide sensitive and specific tools for diagnosing or ruling out CA. These scores can define various levels of amyloid deposition with distinct diagnostic performances.

The mass-to-strain ratio (MSR), a simple metric considering both LV structural and functional properties, has been proposed for differentiating between AL-CA and ATTR-CA [38]. MSR ratio is the most effective discriminator in separating ATTR from AL, though its specificity was lower than the IWT score [38]. Nonetheless, further research is needed to confirm the utility of MSR in this context. In addition, in patients with ATTR-CA, the severity of left ventricular pseudohypertrophy is more pronounced, leading to a lower myocardial strain reserve due to a greater imbalance between pseudohypertrophy and myocardial contractility impairment [38]. This imbalance is attributable to the slower course of ATTR-CA and the lower cardiac toxicity of amyloid fibrils associated with this type of CA [38]. In the diagnostic evaluation of suspected CA in patients with a hypertrophic phenotype, the myocardial strain reserve is employed in conjunction with two echocardiographic scores to identify individuals with ATTR-CA. Ref [38] These scores are the IWT score (which comprises relative wall thickness, E/e′, tricuspid annular plane systolic excursion, longitudinal strain, and systolic apex-to-base ratio) [37] and a simplified version of the IWT score called the AMYLI score, which includes only relative wall thickness and E/e [37] The study found that patients with ATTR-CA had a higher LV mass index, RAS, global longitudinal strain ratio (EFSR), and IWT score, coupled with diminished LV diastolic and biventricular systolic function compared to those with AL-CA [38]. The MSR is more effective than RAS and EFSR in discriminating between ATTR and AL, and its AUC is comparable to the IWT score. An MSR of 11.5 provides 80% specificity and 79% sensitivity for identifying ATTR cardiomyopathy [38]. The MSR score appears to effectively distinguish patients with ATTR-CA from those with confirmed CA, and MSR and IWT exhibit similar diagnostic performance in differentiating those with suspected CA or those with a suspicion of CA and increased wall thickness. Thus, the MSR score is a useful diagnostic tool for identifying the amyloid subtype and requires only the evaluation of two parameters [38].

#### 2.4.2. Cardiac Magnetic Resonance (CMR)

In addition to echocardiography, CMR plays a pivotal role in the diagnostic algorithm of CA. Characteristic CMR findings in CA include diffuse or transmural late gadolinium enhancement (LGE), abnormal gadolinium kinetics, and high extracellular volume (ECV) [5,14]. Recent evidence shows that an LGE pattern can progress from subendocardial to transmural [16], during the course of the disease. Importantly, normal wall thickness does not preclude the presence of abnormal LGE and gadolinium kinetics. However, administration of gadolinium should be performed with caution or avoided altogether in patients with moderate-to-severe renal disease (estimated glomerular filtration rate, eGFR < 30 mL/min/1.73 m^2^) [29], which unfortunately represents a significant number of patients with CA, and thus limits the utility of CMR in this clinical scenario. Native T1 can track ventricular amyloid load, while postcontrast T1 mapping can be used to compute an ECV increase caused by amyloid infiltration. ECV > 0.40% has been shown to be a valuable early diagnostic tool, as well as a prognostic sign in both AL and TTR amyloidosis [5,39,40]. Notably, CMR LGE is more sensitive than echocardiography for detecting cardiac amyloidosis and distinguishes it from other causes of cardiomyopathy, with a sensitivity up to 88% and a specificity up to 92% [29,31]. Consequently, a negative CMR can be safely used to exclude the diagnosis of CA in certain clinical scenarios, while a positive CMR significantly raises the likelihood of CA. Despite the great performance of echocardiography and CMR to diagnose CA, they are considerably limited by their inability to differentiate between subtypes of CA. Further evaluation is needed until a final diagnosis is established.

#### 2.4.3. Bone Scintigraphy

Recent advances in bone scintigraphy and dedicated tracers have turned this imaging modality into the cornerstone of CA evaluation. Bone scintigraphy with technetium 99 m (99 mTc-pyrophosphate [PYP], 99 mTc, 3,3-diphosphono-1,2-propanodicarboxylic acid [DPD], or 99 mTc-hydroxymethylene diphosphonate [HMDP]) should be performed on any patient with suspected CA, either concomitantly with monoclonal protein evaluation to save time, or after a negative protein assessment. The results of bone scintigraphy are interpreted in a semi-quantitative way, with Grade 0 showing no tracer myocardial uptake and normal bone uptake; Grade 1 showing lower myocardial uptake compared to bone level; Grade 2 showing equal myocardial and bone uptake; and Grade 3 showing greater myocardial uptake compared to bone. In addition, a quantitative analysis of scintigraphy results determining the ratio of tracer uptake between the heart (H) and the contralateral half of the lung (CL), referred to as the H/CL ratio, can be applied, with values > 1.5 considered positive [41,42]. Importantly, bone scintigraphy should always be combined with SPECT imaging, to avoid false positive results due to blood pooling in the heart. In addition, the result of a radionuclide scan should always be interpreted in combination with the results of monoclonal protein screening, while false negative results can be observed, and further evaluation is required if clinical suspicion is high in the case of a negative scan. Ultimately, a significant number of patients with AL (ranging from 10 to 40%), present some degree of cardiac uptake; myocardial scarring can result in decreased uptake, but mitral annular and aortic valve calcifications can result in higher uptake creating additional diagnostic pitfalls.

Based on the results of bone scintigraphy and monoclonal protein assessment, four clinical scenarios can occur: (1) negative scintigraphy for cardiac uptake and negative monoclonal proteins, (2) positive scintigraphy (Grade 2–3 uptake) for cardiac uptake and negative monoclonal proteins, (3) negative scintigraphy for cardiac uptake and positive monoclonal proteins, and (4) positive scintigraphy for cardiac uptake and positive monoclonal proteins. The most important contribution of bone scintigraphy in the diagnosis and subtyping of CA is that in the case of positive myocardial uptake (Grade 2–3) and negative monoclonal protein assessment (clinical scenario 2), a diagnosis of ATTR can be made without the need of any further invasive procedures. If some degree of myocardial uptake is recorded (Grade 1) and the monoclonal protein assessment is negative, tissue biopsy is inevitable for the final diagnosis. Accordingly, any other clinical scenario requires tissue biopsy to confirm and differentiate the type of CA. An outline of the diagnostic approach of CA is shown in Figure 1.

## 3. Tissue Biopsy

In any scenario other than negative monoclonal protein assessment and Grade 2–3 myocardial uptake, which establishes the diagnosis of ATTR, the definite diagnosis and subtyping of CA requires tissue biopsy [5,14]. In localized amyloidosis, tissue samples must be taken from the affected organ or tissue; however, in the case of systemic amyloidosis, they may also be taken from abdominal fat, rectum, bone marrow, or salivary glands [6,43,44]. Commonly, on less invasive surrogate sites, a biopsy is performed first (fat biopsy, minor salivary glass, bone marrow), but the most sensitive biopsy site is that of affected organs (heart and/or kidney) [5,14]. The likelihood of a successful diagnosis depends on the involvement of the surrogate site as well as the type of amyloid deposits. A negative finding at a surrogate location, however, does not rule out the diagnosis, and a biopsy of the affected organ should be done if clinical suspicion remains strong [2].

### 3.1. Tissue Amyloid Visualization

Current guidelines suggest abdominal fat aspiration and bone marrow biopsy, and Congo red staining for the diagnosis of AL. Congo red staining highlights the amyloid as a red or salmon-pink (but partly also yellow orange) [7] substance that exhibits a distinctive apple-green birefringence under polarized light. In hematoxylin and eosin staining, massive amyloid deposits are visible as eosinophilic and amorphous masses, whereas modest and initial amyloid deposits, either interstitial or within artery walls, are challenging to see. Amyloids cannot be correctly classified based on the morphology of Congo red staining. Identification of the amyloid subtype is accomplished by mass spectrometry-based analysis of the tissue, which is the gold standard, with a sensitivity of 88% and specificity of 96% [2]. Some experienced centers also apply immunohistochemistry or immunogold immunoelectron microscopy for the identification of the amyloid subtype. Amyloidosis subtyping with mass spectrometry is essential for proper treatment.

Identification of the amyloid subtype is particularly important, since there are cases where a monoclonal protein is detected and ATTR cannot be excluded from imaging, as Congo red staining alone is insufficient.

### 3.2. Fat Pad Biopsy/Abdominal Fat Aspiration

Fine-needle fat aspiration has been incorporated in the diagnostic algorithm of CA, since it can reliably document amyloid deposits and, in combination with imaging characteristics, can indicate cardiac involvement [2]. Under optimal imaging circumstances, the stated sensitivity is 85.1% and the reported specificity is 97.1% [45]. Both abdominal fat pad excisional biopsy (FPEB) and abdominal fat fine-needle biopsy have been used to identify and classify CA. Their major benefits are simplicity, low cost, high patient tolerance, and lack of severe complications [2]. In patients with suspected AL-CA, abdominal fat fine-needle aspiration biopsy has a high specificity (100%) and sensitivity, with positive findings for amyloid in as many as 84% of cases, but it is much less sensitive for ATTR (ATTRv’s positivity in 45% and ATTRwt’s approximately 15%) amyloidosis [2]. Fine-needle aspiration biopsy can produce better quality material for amyloid typing than FPEB. Depending on the size of the sample, FPEB’s sensitivity for AL amyloidosis ranges from 50% for biopsies less than 700 mm^3^ to 100% for biopsies larger than 700 mm [2,46]. It would be ideal for the initial surgical sample to have a size of at least 1400 mm3 since the fat biopsy can occasionally be divided for other studies, such as immunofluorescence, mass spectrometry, and electron microscopy.

### 3.3. Rectal Biopsy

Rectal biopsy has a reported sensitivity of up to 85% and, in combination with fat pad biopsy, has been the most frequently surrogate biopsy site [47]. Regarding amyloid deposits localization, these are usually found in the muscularis mucosae and submucosa, necessitating the acquisition of an in-depth biopsy sample, to avoid missing the diagnosis [48]. However, technical issues, patient discomfort and significant, though rare, complications, such as bleeding and perforation, have limited the application of rectal biopsy as a first-line choice of surrogate site.

### 3.4. Bone Marrow Biopsy

Bone marrow biopsy must be performed on all patients with AL. Core biopsy is the preferred method for the detection of amyloid deposits, while amyloid deposition can be present in only 50–60% of cases [49]. However, the combination of bone marrow biopsy with fat biopsy will return positive results in 90% of cases with systemic AL [50]. Notably, for amyloid subtyping, amyloid seen only in the bone marrow is not enough for diagnosis, since amyloid deposits have been reported in patients with either wild type or hereditary ATTR [51].

### 3.5. Salivary Gland Biopsy

Salivary gland biopsy involves the removal of one or more minor salivary glands via a small incision in the labial mucosa adjacent to the mandibular canine tooth. Temporary complications such as paresthesia and local swelling may arise in roughly 10% of cases [52]. Salivary gland biopsy is characterized by high sensitivity and specificity for the diagnosis of AL, while it can also detect some forms of hATTR [52,53].

## 4. The Role of Endomyocardial Biopsy

Contemporary developments in non-invasive imaging techniques in combination with surrogate site biopsy, have led to significantly reduced EMB procedures performed for the diagnosis of CA. Nonetheless, EMB remains the gold standard method for CA diagnosis, and the last resort when all other diagnostic approaches have failed to establish a diagnosis of CA [5,14]. Consequently, refining the indications of EMB, as well as redefining the existing process in the diagnosis of CA, is essentially needed [54]. Endomyocardial biopsy should be performed if other tissue biopsy does not confirm amyloid and: (1) high clinical suspicion of cardiac amyloidosis in a patient with a monoclonal protein by immunofixation electrophoresis (IFE) and/or an abnormal sFLC K/L; (2) high clinical suspicion for cardiac amyloidosis despite negative or ambiguous PYP scan results; (3) not available cardiac scintigraphy; or (4) rare cases where concomitant ATTR and AL-CA are suspected [54,55] (Table 2).

The diagnostic accuracy of ΕΜΒ has greatly improved due to major advances in equipment and histological analysis techniques. According to current clinical practice standards, at least four pieces of cardiac tissue must be collected [54,55]. EMB is most often carried out in the right ventricle (RV) due to easier access, either via the femoral or internal jugular vein [56]. Tissue is usually collected from the interventricular septum, which is characterized by abundant amyloid deposits, and carries the reduced risk of free wall rupture. Left ventricle EMB is another option, which has shown better diagnostic accuracy in cardiomyopathies compared to RV EMB but is used to a lesser extent due to difficult access [56]. In addition, LV EMB could perform better in detecting early sub-endocardial histological alterations. Both procedures carry risk for significant complications, with the risk of thromboembolism being higher for the LV EMB, and the risk of free wall rupture being higher in the right ventricle [56]. Moreover, extreme caution should be taken for patients with preexisting left bundle branch block (LBBB) undergoing RV EMB, since tissue manipulation and sampling can cause right bundle branch block (RBBB) and AV block, resulting in complete block and the need of a permanent pacemaker. However, LV EMB has a similar risk of complications compared to RV EMB when conducted in facilities with experience^,^ [30,56,57].

Amyloid deposits are most frequently found in the interstitial space, which are seen in a cross-sheet structure [2]. The amyloid deposits in AL amyloidosis are predominantly pericellular and reticular, frequently extending to more than 40% of the myocardial tissue, and may contain inflammatory infiltrates, primarily T-lymphocytes and macrophages, which contribute to tissue damage [41,42,44,45,46,58]. Contrarily, amyloid deposits in ATTR amyloidosis often show two basic patterns: nodular deposits together with diffuse interstitial deposition (pattern A), or thin interstitial and vascular deposits (pattern B). So far, type B fibrils have been observed in ATTRv, whereas type A fibrils may be present in either ATTRv or ATTRwt [2,43].

EMB diagnostic accuracy has been improved by the incorporation of immunohistochemistry and polymerase chain reaction (PCR) in conventional histologic examination and the use of emerging imaging modalities to guide cardiac sampling, while reducing the likelihood of serious consequences [54]. Importantly, the accuracy of the diagnosis is significantly impacted by the quantity and size of tissue fragments collected [30,55]. The Association for European Cardiovascular Pathology guidelines provide recommendations for the number of biopsy samples, with four or more samples yielding a sensitivity and a specificity of about 100%, while one sample establishes a diagnosis in only 20% of the suspected cases [54,55]. For additional focused analyses, three or four more formalin-fixed tissue specimens, as well as one or two snap-frozen or RNA-later-treated tissue samples, should be collected [31]. A sample’s diameter of 1–2 mm is acceptable [54,56] to be examined under light microscopy after routine or specialized histological staining or immunohistochemical staining. Electron microscopy may be used but it needs a specialized sample preparation and is not widely available.

EMB has low sensitivity in some instances and there is a non-negligible risk of major complications, particularly in centers with little experience [55,57]. These highlight the imperative need for proper identification of patients who will actually benefit from such an invasive procedure. It is worth noticing that, even if no evidence of amyloid is detected in the samples, a diagnosis of CA cannot be completely ruled out. This is because amyloid deposits have a diverse distribution throughout the myocardium. They can be disseminated and pericellular, but they can also be nodular and patchy [4], with certain areas of the myocardium having no amyloid deposits at all. When individuals have hereditary amyloidosis in addition to monoclonal gammopathy and the systemic type of amyloidosis, the diagnosis may not be simple to establish. In 3–10% of cases, this occurrence has been documented as nodular [30,50,51]. The value of EMB varies depending on the patient characteristics since the accuracy of diagnosis is greatly influenced by the selection of the patient and the number of tissue samples [54].

## 5. Conclusions

The appropriate diagnosis and subtyping of CA is frequently missed or delayed due to its vague presentation, clinical overlapping, and diagnostic pitfalls. The most important factor for timely diagnosis is increased clinical suspicion, especially in certain clinical scenarios. If CA is the working diagnosis certain steps should be taken. Appropriate imaging with echocardiography or CMR can provide significant evidence for the diagnosis of CA. Importantly, all patients should undergo monoclonal proteins assessment, with these results significantly determining the steps to follow. A negative monoclonal protein assessment will lead to a non-invasive algorithm which, in combination with positive cardiac scintigraphy, can establish a diagnosis of ATTR-CA. The latter is the only clinical scenario in which the diagnosis can be established without the need of biopsy. However, if the imaging results are negative but the clinical suspicion remains high, a myocardial biopsy should be performed. In the case of the presence of monoclonal protein, an invasive algorithm is followed, first by surrogate site sampling and then by myocardial biopsy if the results are inconclusive or prompt diagnosis is needed. The role of endomyocardial biopsy, even though limited by current advances in other techniques, is highly valuable in selected patients and is the only method to reliably establish a diagnosis in challenging cases.

## Figures and Tables

**Table 1:** Red flags of systemic amyloidosis with possible cardiac involvement.

Both Subtypes of CA	AL-Subtype	ATTR-Subtype
Low voltage on ECG and thickening of the septum/posterior wall > 12 mm	HFpEF in combination with nephrotic syndrome	White male age ≥ 65 with HFpEF in combination with history of CTS and/or spinal stenosis
Thickening of RV free wall or valves	Macroglossia and/or periorbital purpura	African American age ≥ 60 with HFpEF without a history of HTN
Intolerance of beta blockers or ACE inhibitors	Orthostatic hypotension	New diagnosis of HCM in an elderly patient
Low normal BP in patients with a previous history of HTN	Peripheral neuropathy	New diagnosis of low-flow, low-gradient aortic stenosis in an elderly patient
History of bilateral CTS, often requiring surgery	MGUS	Family history of ATTRm amyloidosis

Abbreviations: ECG: electrocardiography; RV: right ventricle; ACE: angiotensin converting enzyme; HTN: hypertension; CTS: carpal tunnel syndrome, HFpEF: heart failure with preserved ejection fraction; MGUS: monoclonal gammopathy of undetermined significance; HCM: hypertrophic cardiomyopathy.

**Table 2:** Indications of Endomyocardial Biopsy in Cardiac Amyloidosis.

Indications of EMB in a Patient with Suspected CA
1. Cases in which surrogate tissue biopsy does not confirm the presence of amyloid and:
a. high clinical suspicion for cardiac amyloidosis despite negative or ambiguous PYP scan results;
b. high clinical suspicion of cardiac amyloidosis in a patient with positive monoclonal protein assessment by immunofixation electrophoresis (IFE) and/or an abnormal sFLC K/L;
c. not available cardiac scintigraphy.
2. Cases in which surrogate tissue biopsy does not allow subtyping of amyloid and:
a. abnormal serum free light chain assay and positive cardiac scintigraphy;
b. patients with plasma cell dyscrasia and ambiguous imaging results.
3. For cases in which proper diagnosis is of great importance for timely treatment initiation, cardiac biopsy can be performed to avoid delays.
4. Rare cases where concomitant ATTR and AL-CA are suspected.

Abbreviations: EMB: Endomyocardial biopsy; CA: Cardiac amyloidosis.

## Data Availability

Not applicable.
